# The relationship between childhood sleep, emotional intelligence and Body Mass Index in school aged children

**DOI:** 10.3934/publichealth.2025006

**Published:** 2025-01-09

**Authors:** Eftychia Ferentinou, Ioannis Koutelekos, Evangelos Dousis, Eleni Evangelou, Despoina Pappa, Maria Theodoratou, Chrysoula Dafogianni

**Affiliations:** 1 Faculty of Nursing, University of West Attica, Athens, Greece; 2 School of Social Sciences, Hellenic Open University, Patras, Greece

**Keywords:** childhood obesity, sleep, emotional intelligence, childhood, body mass index

## Abstract

Sleep duration and quality have been increasingly recognized as critical determinants of childhood obesity risk, with insufficient sleep linked to disruptions in appetite-regulating hormones and unhealthy weight gain trajectories. Emotional intelligence, which involves recognizing, understanding, and managing one's own emotions as well as those of others, has garnered attention for its potential impact on VARIOUS aspects of health and well-being, including weight management. Moreover, childhood obesity remains a significant public health concern worldwide, with multifaceted factors contributing to its prevalence and persistence. Research is starting to reveal how sleep patterns and emotional intelligence (ΕΙ) influence children's weight status. This study aims to investigate the relationship between childhood sleep patterns, EI, and body mass index (BMI) in school-aged children. Utilizing a sample of 614 children, aged 8–12 years (mean age 10.0 y), data on emotional intelligence scores, sleep duration and quality, and BMI measurements were collected and analyzed. The results reveal significant correlations among these variables, indicating that emotional intelligence may play a crucial role in both sleep patterns and BMI outcomes in children (*Mean* = 3.53, *SD* = 0.51 in total sample; *Mean* = 3.53, *SD* = 0.51 in overweight/obese). Specifically, higher emotional intelligence scores are associated with better sleep quality and duration, as well as healthier BMI levels (*p* ≤ 0.001). These findings underscore the importance of considering emotional well-being and sleep hygiene in the context of childhood obesity prevention and intervention efforts. Further research is needed to elucidate the underlying mechanisms driving these relationships and to develop targeted strategies for promoting emotional intelligence and healthy sleep habits in school-aged children.

## 1. Introduction

Sleep in childhood is influenced by both biological elements and behavioral habits [1]. Important components of pediatric sleep well-being include sleep duration, quality, timing, efficiency, and related behaviors [2]. Sleep problems in children can be significant indicators or predictors of psychiatric issues such as depression and developmental disorders. Previous studies on children have found that inconsistencies in sleep patterns are linked to later mood disorders and the onset of anxiety disorders [3]. The physical advantages of good sleep habits have been linked to a more advantageous pattern of responding to stress [4]. In addition to sleep duration, the timing of sleep patterns might also influence the risk of obesity. Given that by 2030, the global prevalence of childhood overweight and obesity is projected to reach 30.0% (34.2% for boys and 27.4% for girls), it is important to understand the various biological and behavioral mechanisms underlying this correlation [5]. Sleep can influence the likelihood of developing obesity through both physiological and behavioral pathways. For instance, sleep disturbances can lower leptin levels, a hormone crucial for fat storage regulation and appetite suppression, thereby impairing hunger regulation [5]. Additionally, preschoolers who experience poor sleep tend to consume higher amounts of sugar and carbohydrates [6]. Studies have also shown that inadequate sleep duration during early childhood is associated with a higher risk of developing overweight or obesity later in life [7]. Sleep issues and poor sleep quality are more common in contexts where pediatric obesity and high cumulative risk factors are present [8]. In such circumstances, poor sleep health in childhood may heighten the risk of later overweight or obesity, particularly following early exposure to multiple risk factors [7]. Considering both biological and behavioral pathways, it is evident that sleep and overweight/obesity in children are closely linked. However, further research is needed to explore these relationships comprehensively over time [9].

Sleep difficulties significantly impact children's daily functioning and overall well-being. They can lead to daytime drowsiness, lack of focus, impaired memory, poor impulse control, behavioral issues, academic underachievement, and learning difficulties. Additionally, sleep problems are linked to obesity, diabetes, oppositional defiant disorder, depressive disorders, and, during adolescence, substance abuse, depression, and suicidal thoughts [10]. Likewise, Voci [11] discovered that sleep disruptions such as delayed sleep onset, insufficient sleep duration, and frequent awakenings at night are frequently associated with migraines in children, particularly in those with more severe headaches and less responsive to acute treatments. According to Keefer [12], individuals with high emotional intelligence (EI) exhibit several pathways leading to better health outcomes. They tend to confront stressors directly rather than avoiding or distracting themselves from them, thereby shortening the duration of stressful situations. Socio-emotional skills include capabilities, traits, and qualities crucial for personal achievement and effective social interaction. These skills encompass behavioral tendencies, internal states, task approaches, and the regulation of behavior and emotions [12]. Additionally, they include an individual's beliefs about themselves and the world, which shape their relationships with others. Consequently, individuals with higher EI are less likely to engage in passive rumination or substance use to numb emotions, opting instead for beneficial coping mechanisms when dealing with health challenges or long-term illnesses. Despite the established relationship between EI and health in adults and young people, its replication in children has been limited until now [12].

## 2. Material and methods

### 2.1. Procedure

Between November 2021 and November 2023, individuals participated in a cross-sectional study by voluntarily completing an anonymous questionnaire. The sample comprised 614 children aged 8–12 years, recruited from elementary schools ranging from third to sixth grade and their parents. This convenience sample was drawn from elementary schools across the Attica region. We randomly selected elementary schools in Attica and communicated with the principals and Boards of Teachers. Schools that agreed to participate were included in the study. The questionnaire included an informed consent form for parents to sign. Out of the 28 schools approached, 12 agreed to participate. From the 2320 questionnaires distributed, 925 were returned, and 614 of these were correctly completed and included in the study.

Sociodemographic data were collected through the questionnaire, which included sections for parents to provide information on their child's age, gender, grade, and other relevant background details such as family income, parental education levels, and household composition. This information helped contextualize the study's findings and allowed for the analysis of potential sociodemographic influences on the study outcomes. Specific cutoffs for BMI categories were used based on self-reported height and weight data provided in the questionnaire. The cutoffs were established according to standardized growth charts by Cole et al. [13]. These cutoffs were chosen to ensure consistency and reliability in categorizing children's BMI into underweight, normal weight, overweight, and obese. The use of standardized cutoffs facilitated comparisons with other studies and helped in accurately assessing the relationship between BMI, sleep, and emotional intelligence in the study population.

#### 2.1.1. Inclusion/exclusion criteria

The inclusion criteria for the study were as follows: children aged 8–12 years old, enrolled in third to sixth grade, attending elementary schools in the Attica region, with written informed consent from their legal guardians, and who voluntarily agreed to participate and complete the questionnaire. The exclusion criteria were children younger than 8 or older than 12 years, not enrolled in third to sixth grade, attending schools outside the Attica region, without written informed consent from their legal guardians, returning incomplete questionnaires, or not voluntarily participating in the study.

### 2.2. Aim

This study aims to investigate the relationship between sleep quality, trait emotional intelligence (EI), and body mass index (BMI) in children aged 8–12 years.

### 2.3. Ethics approval of research

The research received approval from the Ethics Committee of the University of West Attica (No. 93329 – 13/11/2020) and the scientific councils of the Ministry of Education and Religious Affairs in Athens, Greece (No. 32/25–06–2021). Approval was also obtained from the school principal and the Board of Teachers. Due to global pandemic restrictions, an electronic version of the questionnaire was developed for distribution and collection. Questionnaires were completed using Microsoft Forms via an online platform that manages personal data such as IP addresses. In accordance with GDPR, the research team confirmed that they could not access participants' IP addresses. The questionnaire did not contain any personal data.

### 2.4. Instruments

The research tool consisted of four sections: (1) Sociodemographic questionnaire, which gathered data on gender, age, marital and educational status, place of residence, occupational status, eating habits, and self-reported weight and height. BMI was calculated using reported height and weight. For children, BMI was calculated using growth charts by Cole et al., with cutoff values for underweight, normal weight, overweight, and obesity [13]. (2) Family Affluence Scale (FAS), calculated using four questions to measure family wealth and classify participants into low, moderate, or high affluence [14]. (3) Trait Emotional Intelligence Questionnaire-Child Short Form (TEIQue-CSF), tailored for children aged 8–12 years, comprising 36 items rated on a five-point Likert scale [15]. TEI encompasses emotional self-perceptions and tendencies that facilitate emotional competence in everyday life [16],[17]. The total score provides an overall measure of TEI. (4) Children's Sleep Habits Questionnaire (CSHQ), which assesses sleep-related issues in school-aged children (8–12 years old) [18]–[20]. A total score of ≥41 points indicated potential sleep problems. Parents completed the CSHQ with written consent for their children's participation. Typically, parents completed the CSHQ. Written consent was obtained from all parents for their children's participation in the study.

### 2.5. Statistical analysis

*Means* and standard deviations (*SD*) described quantitative variables, while absolute (n) and relative (%) frequencies described qualitative variables. Parametric tests were conducted based on the central limit theorem and sample size. Student's t-test compared scales between BMI groups of children. Sleep duration was associated with age, gender, and BMI via Student's t-tests and ANOVA, with Bonferroni correction to control for type-I error. Pearson's correlation coefficient (r) examined relationships between quantitative variables. Linear regression analysis identified independent factors associated with emotional intelligence, yielding regression coefficients (β) and standard errors (SE). The interaction term of BMI with the overall CSHQ score was included to examine the impact of sleep problems on emotional intelligence across BMI levels. Significance levels were two-tailed, with statistical significance set at 0.05. Analyses were performed using SPSS 26.0 software.

## 3. Results

### 3.1. Sample characteristics

The sample consisted of 614 children (54.4% girls, mean age 10.0 years, *SD* = 1.5) and their parents/guardians (76.9% women, mean age 41.8 years, *SD* = 5.3). Child and parent/guardian characteristics are provided in Table 1. Among the children, 62.1% had normal BMI, 29.8% were overweight, and 5.9% were obese. Most children (96.9%) were from Greece, with 31.6% attending the third grade. Additionally, 92.2% lived with both parents, and 99.3% of parents were biological parents. The majority of parents (91.9%) were married, and 61.1% had high socioeconomic status.

**Table 1.** Sample characteristics (*n* = 614).

Project			*n* (%)
Children's characteristics	Age (years), *Mean* (*SD*)		10.0 (1.5)
	Gender	Girls	334 (54.4)
		Boys	280 (45.6)
	BMI	Underweight	14 (2.3)
		Normal	381 (62.1)
		Overweight	183 (29.8)
		Obese	36 (5.9)
	Greek nationality	595 (96.9)
	Albanian nationality	19(3.1)
	Class	3^rd^ grade	194 (31.6)
		4^th^ grade	120 (19.5)
		5^th^ grade	135 (22.0)
		6^th^ grade	165 (26.9)
	Living with:	Only mother	41 (6.7)
		Only father	7 (1.1)
		Both parents	566 (92.2)
Parents' characteristics	Age (years), *Mean* (*SD*)		41.8 (5.3)
	Gender	Women	472 (76.9)
		Men	142 (23.1)
	Relationship with child	Biological parent	610 (99.3)
		Step-parent	4 (0.7)
	Socioeconomical status (FAS)	Low	15 (2.4)
		Moderate	224 (36.5)
		High	375 (61.1)
	Highest parental educational level	High school	198 (32.2)
		Technical university	138 (22.5)
		University	133 (21.7)
		MSc	110 (17.9)
		PhD	35 (5.7)
	Married	564 (91.9)
	Divorced	50 (8.1)

### 3.2. TEIQue and CSHQ scores by BMI

Table 2 presents children's scores on the CSHQ and TEIQue, both for the entire sample and by BMI. Underweight/normal children had significantly higher TEIQue scores, indicating greater emotional intelligence, than overweight/obese children. Overweight/obese children had significantly higher scores on *Bedtime resistance*, *Sleep anxiety*, *Night waking*, *Parasomnias*, *Sleep-disordered breathing*, and *Daytime sleepiness*.

**Table 2.** TEIQue and CSHQ scores in the total sample and by children's BMI levels.

Project	Total sample		BMI				*p*
			Underweight/ normal		Overweight/ obese		

	*Mean*	*SD*	*Mean*	*SD*	*Mean*	*SD*	
TEIQue-CSF	3.53	0.51	3.64	0.46	3.35	0.54	<0.001
Bedtime resistance	8.71	2.56	8.49	2.46	9.11	2.69	0.004
Sleep onset delay	1.33	0.61	1.36	0.62	1.28	0.59	0.137
Sleep duration	3.63	1.12	3.64	1.21	3.59	0.94	0.601
Sleep anxiety	6.16	2.21	5.96	2.13	6.51	2.32	0.003
Night wakings	3.77	1.12	3.63	1.06	4.01	1.17	<0.001
Parasomnias	8.27	1.73	8.07	1.47	8.62	2.07	<0.001
Sleep-disordered breathing	3.34	0.77	3.27	0.73	3.46	0.82	0.004
Daytime sleepiness	13.91	3.39	13.66	3.05	14.37	3.89	0.012
Total CASH	46.04	7.93	45.13	7.12	47.67	9.00	<0.001

In Table 3, overweight/obese children had significantly lower sleep duration compared to underweight/normal ones. Also, boys slept significantly less on a daily basis than girls. Age was found to be significantly associated with the duration of sleep. More specifically, after Bonferroni correction, it was found that 11-year-old children slept significantly less than 8-year-olds (*p* = 0.040) and 9-year-olds (*p* = 0.028). Similarly, 12-year-old children slept significantly less than 8-year-olds (*p* = 0.001) and 9-year-olds (*p* < 0.001).

**Table 3.** Duration of sleep by age, gender, and BMI.

Project		Duration of sleep (hours/day)		*p*
		*Mean*	*SD*	
Age	8	9.1	0.9	<0.001^+^
	9	9.1	0.9	
	10	8.9	0.9	
	11	8.8	0.9	
	12	8.7	1.0	
BMI	Underweight/normal	9.0	0.9	<0.001^++^
	Overweight/obese	8.7	0.9	
Gender	Girls	9.0	1.0	0.050^++^
	Boys	8.8	0.9	

Note: ^+^ *ANOVA*; ^++^ Student's *t*-test

In Table 4, Pearson correlation coefficients between the TEIQue-CSF and CSHQ scales are provided for the entire sample as well as separately for underweight/obese and overweight/obese children. Significant negative correlations were found between the TEIQue-CSF and CSHQ scales, indicating that higher levels of children's sleep problems were associated with lower emotional intelligence. The aforementioned results concern all samples as well as each BMI group. Multiple linear regression was conducted with the emotional intelligence score as the dependent variable and children's and parents' characteristics as well as the overall score of the CSHQ scale as independent variables.

**Table 4.** Pearson's correlation coefficients between TEIQue and CSHQ scores in the total sample and by children's BMI levels.

Project		TEIQue-CSF		
		Total sample	Underweight/normal	Overweight/obese
Bedtime resistance	*R*	−0.28	−0.17	−0.38
	*p*	<0.001	0.001	<0.001
Sleep onset delay	*R*	−0.18	−0.15	−0.3
	*p*	<0.001	0.002	<0.001
Sleep duration	*R*	−0.13	−0.17	−0.12
	*p*	0.001	0.001	0.016
Sleep anxiety	*R*	−0.26	−0.15	−0.37
	*p*	<0.001	0.003	<0.001
Night wakings	*R*	−0.26	−0.17	−0.3
	*p*	<0.001	0.001	<0.001
Parasomnias	*R*	−0.3	−0.23	−0.33
	*p*	<0.001	<0.001	<0.001
Sleep-disordered breathing	*R*	−0.18	−0.09	−0.26
	*p*	<0.001	0.081	<0.001
Daytime sleepiness	*R*	−0.28	−0.17	−0.37
	*p*	<0.001	0.001	<0.001
Total CASH	*R*	−0.39	−0.28	−0.48
	*p*	<0.001	<0.001	<0.001

Additionally, in order to examine whether the impact of sleep problems varies according to children's BMI, the interaction term of BMI with the overall score of the CSHQ scale was also included in the analysis. The results of the analysis are presented in Table 5.

**Figure 1.**
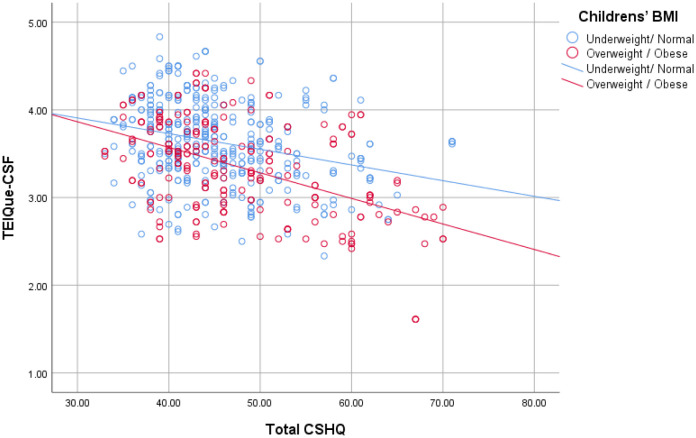
CSHQ and TEIQue-CSF correlation by children's BMI levels.

It was found that age, BMI, gender, socioeconomic status, and sleep problems were significantly associated with children's emotional intelligence. Specifically, as age increases, children's emotional intelligence decreases. Additionally, boys have significantly lower emotional intelligence compared to girls. Children from high socioeconomic backgrounds had significantly higher emotional intelligence compared to children from moderate/low socioeconomic backgrounds. Overweight/obese children had significantly lower emotional intelligence compared to normal-weight children. Moreover, more sleep problems were associated with significantly lower emotional intelligence, especially in overweight/obese children (Figure 1).

**Table 5.** Multiple linear regression results with TEIQue-CSF score as dependent variable.

**Project**	***β* ^+^**	***SE* ^++^**	***p***
Children's age	−0.038	0.013	**0.004**
Children's gender (boys *vs*. girls)	−0.087	0.038	**0.021**
Socioeconomical status (FAS) (high *vs*. low/moderate)	0.147	0.038	**<0.001**
Child living with both parents (yes *vs*. no)	−0.147	0.189	0.435
Parent's age	−0.003	0.004	0.396
Parent's gender (men *vs*. women)	0.001	0.046	0.976
Married parents (Yes *vs*. no)	0.134	0.185	0.469
Highest parental educational level	0.015	0.015	0.309
Children's BMI (overweight/obese *vs*. underweight/normal)	−0.208	0.039	**<0.001**
Total CSHQ^1^	−0.018	0.003	**<0.001**
Interaction term of Children's BMI *Total CSHQ^1^	−0.009	0.005	**0.043**

Note: ^1^analysis was done with centered total CSHQ values for better interpretation ^+^regression coefficient ^++^standard error. The asterisk (*) in the context of Children's BMI * Total CSHQ1 typically represents the inclusion of an interaction term in a statistical model, such as a regression analysis. Interaction Term = Children's BMI × Total CSHQ1.

## 4. Discussion

The aim of this study was to examine the correlation between sleep quality, trait-based emotional intelligence (EI), and body mass index (BMI) in school-aged children. One potential mechanism underlining the observed relationships is the role of the hypothalamic-pituitary-adrenal (HPA) axis in regulating stress responses [21]. Disrupted sleep may lead to dysregulation of the HPA axis, which in turn affects emotional regulation and eating behaviors, contributing to higher BMI. Future research should explore the biological pathways linking sleep, EI, and BMI, including hormonal changes and brain activity patterns. Additionally, longitudinal studies could provide insights into the causal relationships among these variables over time. The findings of this study have significant implications for public health strategies aimed at preventing childhood obesity. Ensuring that children get adequate sleep should be a key component of these strategies. Schools and communities can promote good sleep hygiene through educational programs and by creating environments that support healthy sleep patterns. Public health campaigns could emphasize the importance of sleep for emotional and physical well-being, alongside nutrition and physical activity.

The research aligns with the concerning trend highlighted in the 2022 WHO Regional Report on Obesity in Europe, revealing that approximately one-third of children in the European Region are either overweight or obese [22]. In fact, in our study, 29.8% of participants were overweight and 5.9% were obese. According to Galván, in adolescents, insufficient sleep is linked to less effective emotion regulation, heightened anxiety, increased perception of threats, negative mood, and impaired social interactions [23].

Early intervention is crucial to address persistent sleep issues before they become strongly linked with other behavioral problems like anxiety, depression, or aggression. Literature supports the use of behavioral therapy as the primary treatment approach for children experiencing bedtime difficulties and nighttime awakenings [24]. The National Sleep Foundation recommended that elementary school children (aged 6–13 years) should aim for a sleep duration of 9–11 hours [25]. However, in this study, we found that older participants slept 8.7 hours (*Mean*) and younger participants slept 9.1 hours (*Mean*). This suggests that a significant number of children fail to meet the recommended duration of sleep. Prior research has indicated that during childhood, insufficient sleep duration typically results from bedtime being pushed later, a pattern that tends to shift to even later times as children grow older [25]. This statement agrees with Hosokawa et al., who found that as students advance through the grades, they tend to sleep less [24]. Moreover, in children, inadequate sleep is associated with diminished emotion regulation [23], a result that was also found in this study. In fact, we found that more sleep problems were associated with significantly lower emotional intelligence, but especially in overweight/obese children, where this negative impact was significantly greater. Epidemiological research conducted in Europe and the United States indicates that around one-fourth of children experience daily sleep issues, including insufficient sleep, difficulty waking up, or various sleep disorders such as insomnia, excessive daytime sleepiness, and breathing problems during sleep. It has been observed that the cognitive functioning [26] of school-aged children during the day is influenced not only by diagnosed sleep disorders [27] but also by poor-quality sleep [26]. Findings differed by gender as it was found that male children slept significantly less on a daily basis than female children. Moreover, as far as the duration of sleep is concerned, we found that overweight/obese children sleep 8.7 hours, compared to normal/underweight children who sleep 9 hours. This relationship between increased BMI and shorter duration of sleep in childhood has been established in prior studies [28],[29].

As far as emotional intelligence is concerned, we observed that as age increases, children's emotional intelligence decreases. The adolescent stage is considered a crucial period for identifying the processes that contribute to the development of emotion regulation [30]. This research indicates that poor emotional intelligence in children might correlate with sleep disorders, even after accounting for factors like age, gender, and sleeping patterns; this hypothesis agrees with Takeshima et al. [31]. Specific behavioral interventions to improve sleep and EI in children could include establishing consistent bedtime routines, reducing screen time before bed, and creating a sleep-conducive environment. Cognitive-behavioral therapy (CBT) techniques can help children develop better emotion regulation skills [32], which may in turn improve sleep quality. Programs that integrate sleep education with emotional intelligence training could be particularly effective.

The implications of the findings for different socioeconomic groups are profound. Children from lower socioeconomic backgrounds are more likely to experience both sleep disturbances and lower EI, which can exacerbate issues related to BMI. Interventions should be tailored to address the unique challenges faced by these groups, such as providing resources and support for parents to establish healthy sleep habits and emotional regulation techniques in the home.

In fact, in this study, boys presented significantly lower emotional intelligence than girls. These results provide insight into how emotional self-awareness, crucial for distinguishing oneself from others, may explain gender variations in empathetic abilities during childhood. The association emerging between socioeconomic status and emotional intelligence aligns with our findings, which indicate that children from higher socioeconomic backgrounds exhibit notably greater emotional intelligence in contrast to those from moderate to low socioeconomic backgrounds, resonating with Gruijters' research [33]. Gruijters' study, which drew upon data from 74 countries in the 2018 Program for International Student Assessment, posited that qualities such as self-control, optimism, perseverance, confidence, and a growth mindset are particularly crucial for children from lower-income backgrounds. These traits become essential for overcoming challenges that are more prevalent among less affluent children [33]. Essentially, this suggests that children from more advantaged family backgrounds, characterized by high socioeconomic status, are more likely to develop socio-emotional skills.

## 5. Limitations

Regarding the limitations of our study, data were collected during the lockdown period in which children were required to stay indoors for a prolonged period, while schools remained shut. This situation may have influenced the participants' responses. Moreover, our study is a cross-sectional design, which restricts our ability to infer causality between sleep hygiene, emotional intelligence, and childhood obesity. While significant associations were identified, these findings do not establish a causal relationship. To address this, future research should employ longitudinal study designs and randomized controlled trials to more rigorously explore these relationships. Additionally, the use of self-reported data for assessing sleep hygiene and emotional intelligence introduces potential bias, as participants may provide responses they perceive as socially desirable. Future studies should incorporate objective measures and validated tools to enhance data reliability. Also, the sample was drawn from a specific geographic area, limiting the generalizability of our findings to other populations. Further research with diverse and larger samples is necessary to validate our results and ensure broader applicability.

## 6. Conclusions

In conclusion, our study is the first to shed light on the intricate interplay between childhood sleep patterns, emotional intelligence (EI), and body mass index (BMI) in school-aged children. Through our analysis, we have identified significant correlations among these variables, highlighting the importance of considering sleep quality, emotional well-being, and BMI concurrently in assessing children's overall health and development. In fact, our findings indicate significant associations between sleep hygiene, emotional intelligence, and childhood obesity. However, given the limitations of our study design, it is premature to recommend comprehensive interventions targeting these variables for weight management and overall well-being in school-aged children. Instead, our results underscore the need for more robust research to understand the causal relationships between these variables. Future studies employing designs that support causal analytic techniques are essential. Such research will provide the scientific basis needed to develop interventions targeting the factors contributing to childhood obesity.

Further research is warranted to explore the underlying mechanisms and potential causal relationships between these factors, paving the way for more targeted and effective interventions to support children's holistic development and health outcomes. Concrete actions for practice and policy are suggested, such as implementing school-based sleep education programs, by developing and integrating sleep education curricula into school health programs to teach children the importance of good sleep hygiene, with workshops and informational sessions for parents to educate them on the role of sleep in their children's health and academic performance. Additionally, emotional intelligence (EI) promotion and development of emotional intelligence training into the school curriculum, to help children develop skills such as self-awareness, self-regulation, and empathy. Teachers and school counselors should recognize and support the development of emotional intelligence in students. Mainly, targeted interventions for children identified with sleep disturbances, low EI, or high BMI, including counseling, nutritional guidance, and sleep therapy, such as the implementation of routine screening for sleep problems, emotional well-being, and BMI in school-aged children, to identify those at risk early. Finally, the development of community-based programs that provide resources and support for families to adopt healthier lifestyles. By adopting these recommendations, educators, policymakers, and healthcare providers can work together to support the comprehensive health and development of school-aged children and adjust them based on feedback and new findings.

## Use of AI tools declaration

The authors declare they have not used artificial intelligence (AI) tools in the creation of this article.
